# Distinct genetic alteration profiles of acute myeloid leukemia between Caucasian and Eastern Asian population

**DOI:** 10.1186/s13045-018-0566-8

**Published:** 2018-02-10

**Authors:** Hui Wei, Ying Wang, Chunlin Zhou, Dong Lin, Bingcheng Liu, Kaiqi Liu, Shaowei Qiu, Benfa Gong, Yan Li, Guangji Zhang, Shuning Wei, Xiaoyuan Gong, Yuntao Liu, Xingli Zhao, Runxia Gu, Yingchang Mi, Jianxiang Wang

**Affiliations:** 1 Leukemia Center, Institute of Hematology and Blood Diseases Hospital, Chinese Academy of Medical Sciences & Peking Union Medical College, Tianjin, 300020 China; 2 State Key Laboratory of Experimental Hematology, Institute of Hematology and Blood Diseases Hospital, Chinese Academy of Medical Sciences and Peking Union Medical College, 288 Nanjing Road, Tianjin, 300020 People’s Republic of China

**Keywords:** Acute myeloid leukemia, Mutation, Core binding factor, FLT3-ITD

## Abstract

Racial and ethnic disparities in malignancies attract extensive attention. To investigate whether there are racial and ethnic disparities in genetic alteration between Caucasian and Eastern Asian population, data from several prospective AML trials were retrospectively analyzed in this study. We found that there were more patients with core binding factor (CBF) leukemia in Eastern Asian cohorts and there were different CBF leukemia constitutions between them. The ratios of CBF leukemia are 27.7, 22.1, 21.1, and 23.4%, respectively, in our (ChiCTR-TRC-10001202), another Chinese, Korean, and Japanese Eastern Asian cohorts, which are significantly higher than those in ECOG1900, MRC AML15, UK NCRI AML17, HOVON/SAKK AML-42, and German AML2003 (15.5, 12.5, 9.3, 10.2, and 12%, respectively). And CBFbeta-MYH11 occurred more prevalently in HOVON/SAKK AML- 42 and ECOG1900 trials (50.0 and 54.3% of CBF leukemia, respectively) than in Chinese and Japanese trials (20.1 and 20.8%, respectively). The proportion of FLT3-ITD mutation is 11.2% in our cohort, which is lower than that in MRC AML15 and UK NCRI AML17 (24.6 and 17.9%, respectively). Even after excluding the age bias, there are still different incidence rates of mutation between Caucasian and Eastern Asian population. These data suggest that there are racial and ethnic disparities in genetic alteration between Caucasian and Eastern Asian population.

**Electronic supplementary material:**

The online version of this article (10.1186/s13045-018-0566-8) contains supplementary material, which is available to authorized users.

Acquired genetic abnormalities play an essential role in leukemogenesis and are one of the most important prognostic factors in acute myeloid leukemia (AML). To investigate whether there are some distinctions in genetic alteration profiles among different human races, data from several prospective AML trials were retrospectively analyzed. The ratio of core binding factor (CBF) leukemia in our cohort (ChiCTR-TRC-10001202) is 27.7%. Similarly, the ratios are 22.1, 21.1, and 23.4%, respectively, from Chinese, Korean, and Japanese, the other three Eastern Asian AML cohorts [1–3]. However, the CBF leukemia constitutes 15.5, 12.5, 9.3, 10.2, and 12% of patients, respectively, in ECOG1900, MRC AML15, UK NCRI AML17, HOVON/SAKK AML-42, and German AML2003(Additional file 1: Table S1) [4–8]. All of the latter cohorts are from European or American countries. Therefore, CBF leukemia occurs more frequently in Eastern Asian countries than in European and American countries. Meanwhile, the proportion of NPM1 is 15.9 and 13.3%, respectively, in our cohort and another Chinese one [3] (Additional file 1: Table S2). The proportions of FLT3-ITD mutation are 11.2 and 13.0%, respectively, in our cohort and another Chinese one [3] (Additional file 1: Table S3). Whereas, NPM1 mutation ratios are 27.9, 29.0, and 33.0%, respectively, in MRC AML15, UK NCRI AML17, and German AML2003. FLT3-ITD mutation occurs at 24.6 and 17.9%, respectively, in MRC AML15 and UK NCRI AML17 (Additional file 1: Table S2 and S3) [5, 6, 8]. These data demonstrate that NPM1 and FLT3-ITD mutations are less common in Chinese AML patients in comparison with cases from Europe. It indicates that there are some differences in the frequencies of genetic alteration between Caucasian and Eastern Asian population.

The bias of these comparisons result from the fact that patients from Chinese and Korean cohorts are younger than those from European and American cohorts. To eliminate this bias, we only compared the molecular mutation constitution from patients younger than who were 40 years old in MRC AML10 and AML12 [9–12]. As shown in Table 1, all of the molecular mutations, except FLT3-TKD, are significantly different between MRC cohorts and ours. After excluding the age bias, the ratio of FLT3-ITD mutation is still higher in MRC cohorts, which is similar to the results based on the entire cohorts. And there are more DNMT3a mutations and less CEBPA mutations in MRC cohorts.

**Table 1:** Molecular mutation ratios of patients younger than 40 years old in MRC AML10 and AML12 and our cohort

	MRC AML10 and AML12				China1^a^				*P* value (MRC vs. China1)
	Age range, years	MUT, no.	WT, No.	MUT/total, %	Median age (range), years	MUT, no.	WT, No.	MUT/total, %	
FLT3-ITD [9]	15–34	68	222	23.4	36 (15–55)	66	522	11.2	< 0.001
FLT3-TKD [10]	15–39	46	412	10.0	36 (15–55)	51	534	8.72	0.464
DNMT3a [11]	15–39	71	287	19.8	36 (15–55)	38	348	9.84	< 0.001
CEBPA [12]	15–39	49	524	8.6	36 (15–55)	116	442	20.8	< 0.001
CEBPA double [12]	15–39	33	540	5.8	36 (15–55)	75	483	13.4	< 0.001
CEBPA single [12]	15–39	16	557	2.9	36 (15–55)	41	517	7.3	< 0.001

*Abbreviations*: *WT* wild type, *MUT* Mutant

^a^Data from our cohort (ChiCTR-TRC-10001202)

In order to further confirm that these differences were not due to the onset of age, we analyzed the data in patients older than 40 or 50 years old from Chinese and Japanese trials and compared with Caucasian data. As shown in Additional file 1: Table S4, the frequency of FLT3-ITD in patients older than 40 years old from Chinese trial is only 11.5% which is still significantly lower than that in MRC AML15 and UK NCRI AML17 trial (24.6 and 17.9%, respectively) [5, 6]. There are 24.4 and 18.7% CBF leukemia in patients older than 40 or 50 years old of Chinese and Japanese trials, respectively. They are all significantly higher than those from Caucasian trials, except that the difference between Japanese trials and ECOG1900 did not reach the threshold of statistical significance (Additional file 1: Table S5). NPM1 mutation occurs in 23.6% patients older than 40 years old from Chinese trial. It is still lower than Caucasian data, although there is only statistically difference in comparison with German AML2003 (*P* = 0.005) (Additional file 1: Table S6). These data suggest that AML patients older than 40 years old still had distinctions in frequencies of CBF and FLT3-ITD alterations, which is similar to the difference among the entire cohorts.

As we know, CBF alteration comprises CBFbeta-MYH11 and AML1-ETO fusion genes. Therefore, we finally compared and contrasted the CBF leukemia constitution between Eastern Asian and Caucasian trials. As shown in Table 2, CBFbeta-MYH11 occurred in 50.0 and 54.3% of CBF leukemia in HOVON/SAKK AML- 42 and ECOG1900 trials, respectively [4, 7]. There are only 20.1 and 20.8% CBFbeta-MYH11 in Chinese and Japanese trials, respectively [13], which are significantly lower than Caucasian data. We also calculated the frequencies in older patients in Eastern Asian trials. The CBFbeta-MYH11 ratios are 25.9 and 20.2% in AML patients older than 40 or 50 years old in Chinese and Japanese trials, respectively, which are also significantly lower than that in HOVON/SAKK AML- 42 and ECOG1900 trials and similar to the results of the entire cohort analysis (Table 2). These data indicate that both CBF leukemia frequencies and CBF leukemia constitutions are distinct between Eastern Asian and Caucasian trials, not only in the entire cohorts but also in post hoc analysis after excluding the age bias. We followed the outcome of all these cohorts after we found different mutation landscape. But we only showed, but did not compare the outcome between these cohorts since it is too complicated to compare the outcomes when patients received different therapies (Additional file 1: Table S7).

**Table 2:** CBF leukemia constitution in Japanese and Chinese cohorts against European and American cohorts

	China1^a^	JALSG AML95, 97, and 201 [13]	China1^a^	JALSG AML95, 97, and 201 [13]	HOVON/SAKK AML- 42 [7]	ECOG1900 [4]
Median age, years	36	–	46	–	49	48
Age range, years	15–55	15–64	40–55	50–64	17–60	17–60
CBFβ-MYH11, no.	33	107	15	34	44	57
AML1-ETO, no.	131	408	43	134	44	48
CBFβ-MYH11 in CBF (%)	20.1	20.8	25.9	20.2	50.0	54.3
*P* value (vs. ECOG1900)	< 0.001	< 0.001	< 0.001	< 0.001	0.553	
*P* value (vs. HOVON/SAKK AML- 42)	< 0.001	< 0.001	0.004	< 0.001		0.553

^a^Data from our cohort (ChiCTR-TRC-10001202)

All these data indicate that the different incidence of patterns of mutation acquisition exists between Caucasian and Eastern Asian population, suggesting that genetic backgrounds have an impact on leukemogenesis. We think that these differences become more and more important and are needed to be further investigated, since many novel drugs, such as FLT3 and IDH2 inhibitors, target on genetic mutations and the effectiveness may depend on patterns of mutation acquisition.

## Additional files

Additional file 1: This file contains Tables S1–S7, including **Table S1.** CBF ratios in European, American, and Eastern Asian cohorts; **Table S2.** NPM1 mutation ratios in European and our cohorts; **Table S3.** FLT3-ITD mutation ratios in European and our cohorts; **Table S4.** FLT3-ITD mutation ratios in older patients from Chinese cohort against European and American cohorts; **Table S5.** CBF leukemia ratios in older patients from Japanese and Chinese cohorts against European and American cohorts; **Table S6.** NPM1 mutation ratios in older patients from Chinese cohort against European and American cohorts; and **Table S7.** Outcomes in European, American, and Eastern Asian cohorts. (PDF 568 kb)

## Abbreviations

AML: Acute myeloid leukemia
CBF: Core binding factor
MUT: Mutant
WT: Wild type
