# Supplementary material for: Distinct genetic alteration profiles of acute myeloid leukemia between Caucasian and Eastern Asian population
Source: J Hematol Oncol. 2018 Feb 10;11:18. doi: 10.1186/s13045-018-0566-8 (PMC5807853; doi:10.1186/s13045-018-0566-8)
Supplement: Supplementary file 1 — This file contains Tables S1–S7, including Table S1. CBF ratios in European, American, and Eastern Asian cohorts; Table S2. NPM1 mutation ratios in European and our cohorts; Table S3. FLT3-ITD mutation ratios in European and our cohorts; Table S4. FLT3-ITD mutation ratios in older patients from Chinese cohort against European and American cohorts; Table S5. CBF leukemia ratios in older patients from Japanese and Chinese cohorts against European and American cohorts; Table S6. NPM1 mutation ratios in older patients from Chinese cohort against European and American cohorts; and Table S7. Outcomes in European, American, and Eastern Asian cohorts. (PDF 568 kb) [file 13045_2018_566_MOESM1_ESM.pdf]

Table S1-7

Table S1. CBF ratios in European, American, and Eastern Asian cohorts

	China1*	China2[1]	Korea[2]	JALSG AML201[3]	ECOG1900 [4]	MRC AML15 [5]	UK NCRI AML17[6]	HOVON/SAKK AML- 42 [7]	German AML2003[8]
Median age, years	36	37	43	47	48	49	53	49	48
Age range, years	15-55	14-59	15-60	15-64	17-60	0-73	16-72	17-60	16-60
CBF, No.	164	106	81	247	102	408	112	88	142
Non-CBF, No.	427	373	296	774	552	2343	1032	719	1037
CBF/Total, %	27.7	22.1	21.5	24.2	15.6	14.8	9.8	10.9	12.0
P value (*vs*. China1)		0.035	0.029	0.114	<0.001	<0.001	<0.001	<0.001	<0.001
P value (*vs*. China2)	0.035		0.821	0.380	0.005	<0.001	<0.001	<0.001	<0.001
P value (*vs*. Korea)	0.029	0.821		0.289	0.017	0.001	<0.001	<0.001	<0.001
P value (*vs*. JALSG)	0.114	0.380	0.289		<0.001	<0.001	<0.001	<0.001	<0.001

* Data from our cohort (ChiCTR-TRC-10001202)

Abbreviations: CBF, core binding factor.

Table S2. NPM1 mutation ratios in European and our cohorts

	China1*	China2[1]	MRC AML15 [5]	UK NCRI AML17[6]	German AML2003[8]
Median age (years)	36	37	49	53	48
Age range (years)	15-55	14-59	0-73	16-72	16-60
NPM1 Mutant(No.)	89	64	200	320	375
NPM1 Wildtype (No.)	471	416	517	783	760
NPM1 Mutant/Total (%)	15.9	13.3	27.9	29.0	33.0
P value (*vs*. China1)		0.245	<0.001	<0.001	<0.001
P value (*vs*. China2)	0.245		<0.001	<0.001	<0.001

* Data from our cohort (ChiCTR-TRC-10001202)

Table S3. FLT3-ITD mutation ratios in European and our cohorts

	China1*	China2[1]	MRC AML15 [5]	UK NCRI AML17[6]
Median age (years)	36	37	49	53
Age range (years)	15-55	14-59	0-73	16-72
FLT3-ITD (No.)	66	67	206	200
Non-FLT3-ITD (No.)	522	447	630	920
FLT3-ITD /Total (%)	11.2	13.0	24.6	17.9
P value(*vs*. China1)		0.357	<0.001	<0.001
P value(*vs*.China2)	0.357		<0.001	0.014

* Data from our cohort (ChiCTR-TRC-10001202)

Table S4. FLT3-ITD mutation ratios in older patients from Chinese cohort against European and American cohorts

	China1*	MRC AML15 [5]	UK NCRI AML17[6]
Median age (years)	46	49	53
Age range (years)	40-55	0-73	16-72
FLT3-ITD (No.)	27	206	200
Non-FLT3-ITD (No.)	208	630	920
FLT3-ITD /Total (%)	11.5	24.6	17.9
P value(*vs*. China)		<0.001	0.017

* Data from our cohort (ChiCTR-TRC-10001202)

Table S5, CBF leukemia ratios in older patients from Japanese and Chinese cohorts against European and American cohorts

	China1*	JALSG AML95, 97 and 201[9]	ECOG1900 [4]	MRC AML15 [5]	UK NCRI AML17[6]	HOVON/SAKK AML-42 [7]	German AML2003[8]
Median age (years)	46	-	48	49	53	49	48
Age range (years)	40-55	50-64	17-60	0-73	16-72	17-60	16-60
CBF (No.)	58	168	102	408	112	88	142
Non-CBF (No.)	180	732	555	2843	1094	772	1037
CBF/Total (%)	24.4	18.7	15.5	12.5	9.3	10.2	12
P value(*vs*. China)		0.05	0.002	<0.001	<0.001	<0.001	<0.001
P value(*vs*. Japan)	0.107		0.106	<0.001	<0.001	<0.001	<0.001

* Data from our cohort (ChiCTR-TRC-10001202)

Abbreviations: CBF, CBF leukemia; NON-CBF, NON-CBF leukemia.

Table S6. NPM1 mutation ratios in older patients from Chinese cohort against European and American cohorts

	China1*	MRC AML15 [5]	UK NCRI AML17[6]	German AML2003[8]
Median age (years)	46	49	53	48
Age range (years)	40-55	0-73	16-72	16-60
NPM1 Mutant (No.)	53	200	320	375
NPM1 Wildtype (No.)	172	517	783	760
NPM1 Mutant/Total (%)	23.6	27.9	29.0	33.0
P value (*vs*. China)		0.2	0.097	0.005

* Data from our cohort (ChiCTR-TRC-10001202)

Table S7. Outcomes in European, American, and Eastern Asian cohorts

	China1#	China2[1]	Korea [2]	JALSG AML201[3]	ECOG1900 [4]	MRC AML15 [5]	UK NCRI AML17[6]	HOVON/SAKK AML- 42 [7]	German AML2003[8]
Median age (years)	36	37	43	47	48	49	53	49	48
Age range (years)	15-55	14-59	15-60	15-64	17-60	0-73	16-72	17-60	16-60
**Overall**									
CR rate (after two course) (%)	82.1	67.0	77.3	77.9	63.9	78(DA)<br>82(ADE1*)<br>84(FLAG-Ida)<br>81(ADE2*)	82	80.7	65
RFS (%)	55.4(SD)3y<br>66.7(ID)3y	37.9(DA)3y<br>48.8(HAA)3y<br>46.3(HAD)3y	44.8(5y)	41(IDA)5y<br>41(DNR)5y		35(DA)<br>32(ADE1*)<br>45(FLAG-Ida)<br>34(ADE2*)	48(DA60)2y<br>51(DA90)2y	NA	NA
OS (%)	59.3(DA)3y<br>67.7(HAA)3y	42.7(DA)3y<br>44.5(HAA)3y<br>43.5(HAD)3y	40.8(5y)	48(IDA)5y<br>48(DNR)5y	23.7months&（HD）<br>15.7months&（SD）	36(DA)<br>35(ADE1*)<br>44(FLAG-Ida)<br>37(ADE2*)	60(DA60)2y<br>59(DA90)2y	40(ID)5y<br>42(HD)5y	NA
**Cytogenetic favorable risk group**									
CR rate (%)	NA	NA	93.8	91(IDA)<br>96(DNR)	81.3	NA	NA	NA	NA
RFS (%)	NA	NA	48.8(5y)	NA		NA	NA	NA	NA
OS (%)	NA	NA	57.2(5y)	NA		NA	100(DA60)2y<br>87(DA90)2y	64(ID)5y<br>67(HD)5y	NA
**Cytogenetic intermediate risk group**									

CR rate (%)	NA	NA	74.9	79(IDA)<br>76(DNR)	58.7	NA	NA	NA	NA
RFS (%)	NA	NA	47.5(5y)	NA		NA	NA	NA	NA
OS (%)	NA	NA	41.8(5y)	NA		NA	64(DA60)2y<br>65(DA90)2y	45(ID)5y<br>44(HD)5y	NA
**Cytogenetic adverse risk group**									
CR rate (%)	NA	NA	64.9	51(IDA)<br>43(DNR)	51.4	NA	NA	NA	NA
RFS (%)	NA	71.4(DA)3y<br>NA(HAA)3y<br>60.0(HAD)3y	21.6(5y)	NA		NA	NA	NA	NA
OS (%)	NA	41.2(DA)3y<br>7.5(HAA)3y<br>38.4(HAD)3y	14.0(5y)	NA	10.4months&（HD）<br>10.2months&（SD）	NA	28(DA60)2y<br>23(DA90)2y	31(ID)5y<br>30(HD)5y	NA

Abbreviations: CBF, core binding factor. NA: Data are not available in the reports

*: There are two ADE regimen group in that clinical trial.

&: There is only median survival in this report.

#: Data from our cohort (ChiCTR-TRC-10001202)

Reference

1. Jin J, Wang JX, Chen FF, Wu DP, Hu J, Zhou JF, Hu JD, Wang JM, Li JY, HuangXJ, Ma J, Ji CY, Xu XP, Yu K, Ren HY, Zhou YH, Tong Y, Lou YJ, Ni WM, Tong HY,Wang HF, Mi YC, Du X, Chen BA, Shen Y, Chen Z, Chen SJ. Homoharringtonine-based induction regimens for patients with de-novo acute myeloid leukaemia: a multicentre, open-label, randomised, controlled phase 3 trial. Lancet Oncol. 2013;14(7):599-608.
2. Lee JH, Joo YD, Kim H, Bae SH, Kim MK, Zang DY, et al. Cooperative Study Group A for Hematology. A randomized trial comparing standard versus high-dose daunorubicin induction in patients with acute myeloid leukemia. Blood. 2011; 118:3832-41
3. Ohtake S, Miyawaki S, Fujita H, Kiyoi H, Shinagawa K, Usui N, et al. Randomized study of induction therapy comparing standard-dose idarubicin with high-dose daunorubicin in adult patients with previously untreated acute myeloid leukemia: the JALSG AML201 Study. Blood. 2011; 117:2358-65
4. Fernandez HF, Sun Z, Yao X, Litzow MR, Luger SM, Paietta EM, et al. Anthracycline dose intensification in acute myeloid leukemia. N Engl J Med. 2009; 361:1249-59
5. Burnett AK, Russell NH, Hills RK, Hunter AE, Kjeldsen L, Yin J, et al. Optimization of chemotherapy for younger patients with acute myeloid leukemia: results of the medical research council AML15 trial. J Clin Oncol. 2013; 31:3360-8
6. Burnett AK, Russell NH, Hills RK, Kell J, Cavenagh J, Kjeldsen L, et al. A randomized comparison of daunorubicin 90 mg/m2 vs 60 mg/m2 in AML induction: results from the UK NCRI AML17 trial in 1206 patients. Blood. 2015; 125:3878-85
7. Löwenberg B, Pabst T, Vellenga E, van Putten W, Schouten HC, Graux C, et al. Cytarabine dose for acute myeloid leukemia. N Engl J Med. 2011; 364:1027-36.
8. Schaich M, Parmentier S, Kramer M, Illmer T, Stölzel F, Röllig C, et al. High-dose cytarabine consolidation with or without additional amsacrine and mitoxantrone in acute myeloid leukemia: results of the prospective randomized AML2003 trial. J Clin Oncol. 2013; 31:2094-102
9. Yanada M, Ohtake S, Miyawaki S, Sakamaki H, Sakura T, Maeda T,et al. The demarcation between younger and older acute myeloid leukemia patients: a pooled analysis of 3 prospective studies. Cancer. 2013; 119:3326-3333.
